# CAREGIVING ACROSS GENERATIONS AND CULTURES

**DOI:** 10.1093/geroni/igz038.2124

**Published:** 2019-11-08

**Authors:** Valentine Villa

**Affiliations:** UCLA, Los Angeles, California, United States

## Abstract

Utilizing data from the California Health Interview Survey (CHIS) 2009, we examine caregiving activities among foreign born and US born Latinos, and US born Non-Hispanic whites ages 18 to 80 who are caring for an adult age 60 or older. Utilizing OLS regression, we first examine the relationship between race, ethnicity, nativity, and age and the amount of caregiving, types of caregiving, and use of long-term care (LTC) services among the population. Next we examine the health, mental health, and economic status of the three caregiver populations. The results find that middle aged and younger foreign born Latinos provide the greatest amount of care and also present with the greatest amount of economic challenges. US born middle- aged Latino caregivers also provide relatively high levels of care and also are more likely than other caregivers to experience health challenges including diabetes, obesity, depression and social isolation.

